# Many Drugs of Abuse May Be Acutely Transformed to Dopamine, Norepinephrine and Epinephrine In Vivo

**DOI:** 10.3390/ijms221910706

**Published:** 2021-10-02

**Authors:** Paul J. Fitzgerald

**Affiliations:** Department of Psychiatry, University of Michigan, Ann Arbor, MI 48109, USA; pfitz1940@gmail.com

**Keywords:** cocaine, amphetamine, methamphetamine, ephedrine, MDMA, ecstasy, heroin, morphine, oxycontin, fentanyl, buprenorphine, naltrexone, naloxone, DMT, psilocybin, mescaline, LSD, atropine, scopolamine, nightshades

## Abstract

It is well established that a wide range of drugs of abuse acutely boost the signaling of the sympathetic nervous system and the hypothalamic–pituitary–adrenal (HPA) axis, where norepinephrine and epinephrine are major output molecules. This stimulatory effect is accompanied by such symptoms as elevated heart rate and blood pressure, more rapid breathing, increased body temperature and sweating, and pupillary dilation, as well as the intoxicating or euphoric subjective properties of the drug. While many drugs of abuse are thought to achieve their intoxicating effects by modulating the monoaminergic neurotransmitter systems (i.e., serotonin, norepinephrine, dopamine) by binding to these receptors or otherwise affecting their synaptic signaling, this paper puts forth the hypothesis that many of these drugs are actually acutely converted to catecholamines (dopamine, norepinephrine, epinephrine) in vivo, in addition to transformation to their known metabolites. In this manner, a range of stimulants, opioids, and psychedelics (as well as alcohol) may partially achieve their intoxicating properties, as well as side effects, due to this putative transformation to catecholamines. If this hypothesis is correct, it would alter our understanding of the basic biosynthetic pathways for generating these important signaling molecules, while also modifying our view of the neural substrates underlying substance abuse and dependence, including psychological stress-induced relapse. Importantly, there is a direct way to test the overarching hypothesis: administer (either centrally or peripherally) stable isotope versions of these drugs to model organisms such as rodents (or even to humans) and then use liquid chromatography-mass spectrometry to determine if the labeled drug is converted to labeled catecholamines in brain, blood plasma, or urine samples.

## 1. Introduction

Many drugs of abuse, such as cocaine or opium, either occur naturally in plants or can be readily derived from plant-based chemicals [1,2]. This natural occurrence makes it likely that evolving organisms, such as various animals, that consume a diverse range of plants have evolved mechanisms for metabolizing or inactivating these molecules, such as the cytochrome P450 molecules found in the liver (and the brain) [3]. Whereas many drugs are transformed into metabolites with active psychotropic properties, most drugs are not typically thought to be converted to molecules that occur naturally in the body and participate in normal, endogenous signaling pathways.

Aside from their widely varying intoxicating or euphoriant subjective properties, different classes of drugs of abuse share the property of acutely boosting the body’s stress signaling pathways, namely the sympathetic nervous system and HPA axis which use norepinephrine and epinephrine as major output molecules [4,5,6,7]. This results in what can be perceived as unpleasant side effects such as boosting the heart rate, elevating blood pressure, increasing the rate of breathing, elevating body temperature and accompanied sweating, as well as pupillary dilation. While the mechanisms through which drugs such as stimulants, opioids, and psychedelics boost sympathetic and HPA axis signaling are perhaps continuing to be investigated, they are not thought to be actively converted to endogenous signaling molecules such as the catecholamines (dopamine, norepinephrine, epinephrine).

## 2. Hypothesis: A Wide Range of Drugs of Abuse Are Acutely Transformed to Catecholamines In Vivo

This paper puts forth the hypothesis that a broad range of drugs of abuse—stimulants, opioids, psychedelics—are actually acutely converted to catecholamines in the body. This biochemical conversion, which may occur in a wide range of animals as well as in humans (and possibly other organisms), may not only underlie the boosting of sympathetic nervous system and HPA axis signaling described above, but also contribute to the intoxicating or rewarding properties of these drugs (along with their known interaction with monoaminergic or opioid signaling molecules or receptors). This proposed biotransformation to catecholamines is suggested here to occur in addition to the acute conversion of these drugs to their known metabolites (see below). It is also hypothesized that this conversion to catecholamines, perhaps especially by boosting norepinephrine and epinephrine in an ongoing manner through repeated use of drugs, plays a significant role in substance abuse and dependence, including psychological stress-induced relapse of drug seeking behavior. To my knowledge, this overall hypothesis is novel and has not yet been scientifically investigated, but is directly testable in animals or even human subjects, using stable isotope biochemical experiments (see below).

## 3. Proposed Pathways and Published Data Related to the Hypothesis 

### 3.1. Stimulants

**Figure 1:**
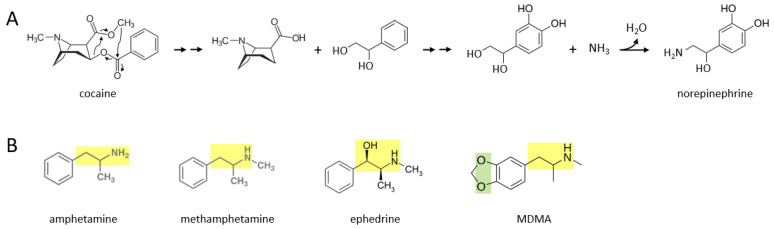
Various stimulants may be acutely converted to catecholamines in vivo. (**A**) Cocaine may be transformed to norepinephrine through a series of proposed chemical reactions. Double arrows indicate two or more reactions. (**B**) Various other stimulants possess moieties (yellow: a two hydrocarbon chain attached to a nitrogen atom; green: two oxygen atoms attached to the benzene ring) that may allow them to be enzymatically converted to dopamine, norepinephrine, or epinephrine.

A number of stimulant drugs—cocaine, amphetamine, methamphetamine, ephedrine, MDMA—may be acutely transformed to catecholamines such as norepinephrine, soon after an individual takes them. Figure 1A shows a proposed series of chemical reactions whereby cocaine is converted to norepinephrine. In this scenario, an unidentified enzyme or enzymes catalyze reactions in which the cocaine molecule is first separated into an alkaloid molecule and a phenolic compound with a two carbon side chain that has two hydroxyl groups attached. During further reactions, the phenolic molecule has two hydroxyl groups attached to its benzene ring. Subsequently, an amine group (where it is possible that the nitrogen atom comes from the original alkaloid molecule) replaces the terminal hydroxyl group on the two carbon side chain, to form norepinephrine. In principle, this norepinephrine molecule could then be converted to epinephrine through the canonical biosynthetic pathway for catecholamines described over 70 years ago [8,9,10,11].

Figure 1B shows the molecular structures of amphetamine, methamphetamine, ephedrine, and MDMA, where each drug consists of a phenolic structure attached to a two carbon side chain plus a nitrogen atom (moiety marked in yellow). It is suggested here that the additional methyl groups attached to this moiety in each drug can be reversibly attached, through currently unidentified enzymatic processes, in the body. Methylation and demethylation have been widely studied with respect to DNA modifications [12], but to my knowledge demethylation has not been suggested to occur frequently in other biochemical pathways involving hydrocarbons, although it has been described industrially [13]. Demethylation of amphetamine or methamphetamine, for example, could yield dopamine, with the addition of two hydroxyl groups to their benzene ring. For ephedrine, these processes could yield epinephrine (or norepinephrine if the terminal methyl group is also removed). MDMA also has two oxygen atoms attached to the other side of its benzene ring, where this moiety (marked in green) may be converted to two hydroxyl groups in vivo. For MDMA, removal of the two methyl groups from the two carbon side chain moiety would yield dopamine. Thus, these five stimulant drugs could be converted to dopamine, norepinephrine, or epinephrine, through yet to be determined enzymatic mechanisms.

### 3.2. Opioids

**Figure 2:**
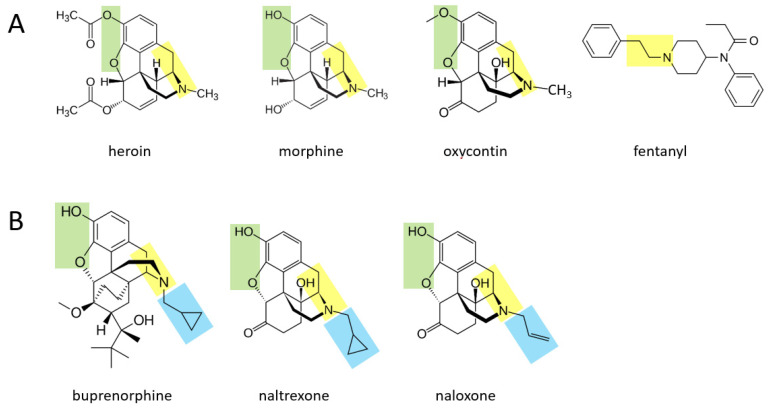
A number of opioids could be transformed to catecholamines in the body. (**A**) These drugs of abuse possess moieties, marked in yellow or green, that may allow them to be converted to catecholamines acutely. (**B**) Compounds used to treat opioid abuse, in contrast, possess an additional moiety (light blue) that may block them from being fully converted to catecholamines and may allow them to serve as a pharmacological blocker of the endogenous pathways acted upon by the four drugs in (**A**).

Various opioid drugs—heroin, morphine, oxycontin, fentanyl—may also be acutely transformed to catecholamines in the body. Figure 2A shows that heroin, morphine, and oxycontin all contain a two hydrocarbon structure attached to a nitrogen atom (moiety shown in yellow), with an additional methyl group attached to the nitrogen atom. These three molecules also contain two oxygen atoms attached to the other side of their benzene ring (green moiety). It is suggested here that through a series of currently unidentified reactions, these three molecules may be converted to catecholamines. In this scenario, the yellow moiety (if the methyl group attached to the nitrogen atom is enzymatically removed) could support the formation of dopamine, where the green moiety would form the two hydroxyl groups on the other side of the benzene ring. If the terminal methyl group is not removed from the yellow moiety, this could support the formation of epinephrine (after an hydroxyl group is added to the carbon atom nearest to the ring). For each of these three drugs, the rest of the molecule would be separated from the forming catecholamine. In the case of fentanyl, this drug could be converted to dopamine if the yellow moiety attached to the benzene ring is separated from the rest of the molecule, and two hydroxyl groups are enzymatically added to the other side of the ring. As for all drugs in this paper, if they are first converted to dopamine, they could in principle then be converted to norepinephrine and subsequently epinephrine through the canonical pathway.

Figure 2B shows three other opioid drugs—buprenorphine, naltrexone, naloxone—that have milder (or no) rewarding properties and are instead used to treat opioid dependence [14]. It is suggested here that these three drugs may also be converted to molecules that resemble catecholamines (based on their yellow and green moieties), but because they have a larger hydrocarbon structure (blue moiety) attached to the nitrogen atom, this may not be enzymatically removable in vivo and the overall, newly formed molecule may serve as a pharmacological blocker of the endogenous pathways acted upon by the four drugs in Figure 2A.

### 3.3. Psychedelics

**Figure 3:**
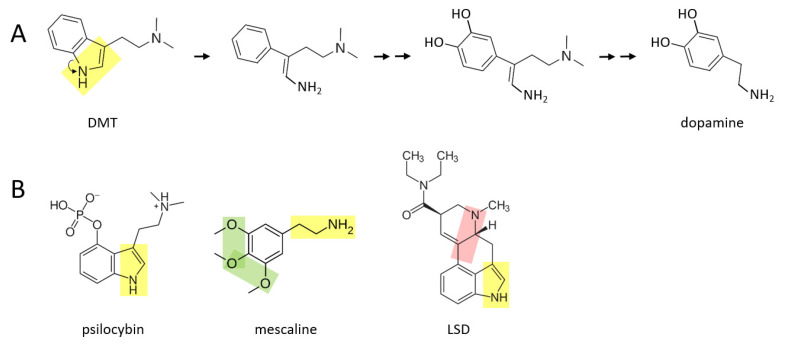
Several psychedelics may be transformed to catecholamines in vivo. (**A**) DMT may be converted to dopamine through a series of proposed chemical reactions. Double arrows indicate two or more reactions. (**B**) Psilocybin and mescaline possess the yellow or green moieties that may allow transformation to catecholamines. LSD is a more complex molecule, and it is suggested here that conversion to catecholamines could occur, in part, either through the yellow or pink moiety.

A number of psychedelic drugs—DMT, psilocybin, mescaline, LSD—may also be converted to catecholamines soon after being ingested by an individual. Figure 3A shows a proposed series of reactions in which DMT is transformed to dopamine. In this scenario, the nitrogen atom “unhooks” from the benzene ring, forming an amine group that is now attached to the benzene ring by a two carbon chain. After this, two hydroxyl groups are enzymatically attached to the other side of the ring. These reactions are followed by enzymatic separation of the additional two carbon chain that has a nitrogen atom with two methyl groups attached, as well as elimination of the double bond in the remaining chain, thereby forming dopamine.

A similar series of reactions may take place for psilocybin (Figure 3B), where an additional phosphate group must be removed from the benzene ring. Mescaline could also form dopamine, based on its yellow moiety, where its green moiety may allow it form a pair of hydroxyl groups on the other side of the benzene ring, from which an extra oxygen group would need to be enzymatically removed. LSD is a larger and more complex molecule than the other three psychedelics shown here. It is suggested here that there may be two means through which LSD could be converted to catecholamines in vivo: (1) by enzymatically unhooking the nitrogen atom from its benzene ring (yellow moiety) and eventually separating the ring and this moiety from the rest of the molecule, or (2) by separating the other nitrogen atom and its two hydrocarbon chain (pink moiety) along with its attached benzene ring, from the rest of the molecule. In either scenario, two hydroxyl groups would have to be added to the other side of the benzene ring. Perhaps only one of these scenarios exists in vivo.

### 3.4. Nightshades

**Figure 4:**
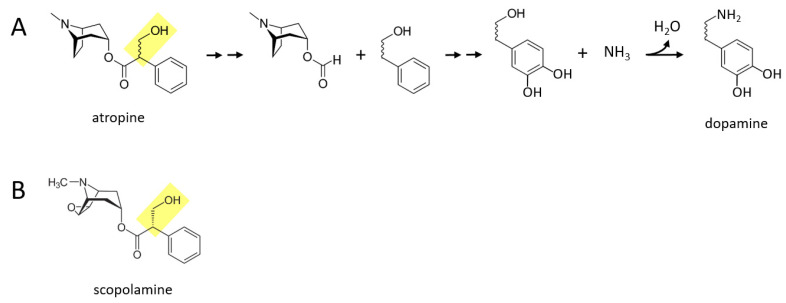
Certain alkaloid drugs found in nightshade plants may also be acutely converted to catecholamines in the body. (**A**) Atropine could be converted to dopamine in a series of proposed reactions. Double arrows indicate two or more reactions. (**B**) Scopolamine, like atropine, contains a moiety (shown in yellow) that may facilitate enzymatic conversion to catecholamines.

Some of the alkaloid drugs from nightshade family (*Solanaceae*) of plants, while not typically being used as drugs of abuse to my knowledge, may also be acutely converted to catecholamines. One possibility is that the poisonous or even lethal properties of some of these drugs is related to their boosting of catecholaminergic signaling, especially norepinephrine and epinephrine. Atropine and scopolamine are two alkaloid drugs found in certain nightshade plants, that are muscarinic cholinergic receptor antagonists [15]. Figure 4A describes a proposed series of reactions in which atropine is converted to dopamine in the body. First, the benzene ring and its attached two hydrocarbon chain with a hydroxyl group (yellow moiety) separate from the rest of the molecule. Next, two hydroxyl groups are attached to the other side of the benzene ring. Finally, the terminal hydroxyl group is replaced with an amine group, forming dopamine. Similar reactions could take place for scopolamine (Figure 4B), where for both drugs, dopamine could in principle be subsequently converted to norepinephrine and epinephrine through the canonical pathway.

A related point regarding cholinergic signaling and neurotransmitter biosynthesis: there may also be a novel series of reactions that convert alcohol (ethanol, a two hydrocarbon chain with a single hydroxyl group) to acetylcholine. In particular, ethanol may combine with trimethylammonium (or perhaps first be converted to ethanolamine) to form choline, which is one of the canonical building blocks of acetylcholine.

## 4. Evaluation of the Hypothesis

The overarching hypothesis put forth here is directly testable with relatively simple and safe biochemistry experiments using stable isotopes. In these experiments, a model organism such as a mouse or a rat (or even a human) is systemically administered a stable isotope version of one of the drugs discussed above, which is engineered to contain an additional neutron in the nucleus of one or more of its atoms. Such compounds are not radioactive and have the same chemical properties as the normal version of the drug. They are commercially available or can be custom engineered by many companies around the world (for example: MilliporeSigma, CDN Isotopes, Charles River Laboratories, Cambridge Isotope Laboratories). Stable isotope alcohol, for example, is already available commercially (ethanol-1-13C, MilliporeSigma, catalog # 324523). Typically, “heavy” carbon (i.e., carbon 13 instead of carbon 12, which is the isotope typically found naturally) or “heavy” hydrogen (deuterium) is substituted for at least one atom of the molecule. This “labeled” drug is then administered to the model organism, and then after a time delay of perhaps an hour or up to a day or two, blood plasma, urine, or brain tissue or cerebrospinal fluid (possibly in conjunction with microdialysis) samples are collected. These samples are then processed using liquid chromatography-mass spectrometry to determine if any of the “heavy” atoms from the labeled drug administration are incorporated into catecholamines (dopamine, norepinephrine, epinephrine). Having an hypothesis regarding which portion of the labeled drug molecule should be preserved in the labeled catecholamine is therefore important to consider in engineering the original labeled drug.

For a carbon 13 labeled drug molecule, for example, one approach to analyzing the data from liquid chromatography-mass spectrometry is to calculate the ratio of carbon 12 to carbon 13 (i.e., C12/C13) in the retention time area plots for each catecholamine, which should be reduced in animals that received carbon 13 stable isotope drug treatment. This raises another important point: half of the animals should receive a “control” injection of the normal, unlabeled version of the drug. That means all animals, whether they receive the labeled or unlabeled version of the drug, would undergo the same injection or infusion stress, and also experience the same period of acute intoxication, where the only difference between groups is the drug labeling. Then, an unpaired two-tailed (or possibly one-tailed) t test could be used to compare the carbon 12 to carbon 13 ratio, for each catecholamine, from all the unlabeled drug animals versus the labeled drug animals. Perhaps a sample size of 10 or more animals per group could be used.

In addition, operating a triple quadrupole mass spectrometer in targeted mode should also reveal if there is a significant peak in the samples for “heavy” versions of any of the three catecholamines. It is suggested here that administering these drugs peripherally to mice or rats (typically intraperitoneally) would still allow the drug to cross the blood–brain barrier, since in humans these drugs are typically administered systemically and still produce intoxicating effects. For brain tissue analysis of animals, whole brain homogenates (including brainstem) could initially be performed, where later, more refined experiments could seek to define which brain circuits and cell types carry out these putative biosynthetic reactions. However, a number of laboratories around the world are already set up to carry out microdialysis analysis of neurotransmitters in a variety of distinct brain regions, which could immediately test the hypothesis put forth here. Of course, it is also possible that putative drug conversion to catecholamines occurs outside the brain as well, in peripheral organs such as the adrenal glands.

## 5. Consequences of the Hypothesis

If many drugs of abuse are acutely converted to catecholamines in vivo, this would have a number of important implications. Not only would this potentially help explain the stimulatory effect of these compounds on the sympathetic nervous system and HPA axis, but it may also contribute to our understanding of their intoxicating or euphoriant properties, since both dopamine and norepinephrine have already been implicated in reward processing in the brain [16,17,18]. It is suggested here that these putative acute effects on boosting catecholaminergic signaling interact with the more established mechanisms of these drugs, which involve modulation of monoaminergic (or opioid) receptors and signaling pathways, to produce their psychotropic qualities (which vary greatly across these classes of drugs). If these drugs tend to boost noradrenergic and adrenergic signaling, this may also play a prominent role in the development and maintenance of substance abuse, since the tone of norepinephrine and epinephrine may tend to remain elevated with repeated drug use and this has been implicated in substance abuse [17]. Acute boosting of catecholamines could also affect psychological stress-induced relapse of drug seeking behavior, as the individual may seek drugs during or after stress because they crave the rewarding properties of boosting dopamine or norepinephrine, and this could also be a means of replenishing depleted cellular stores of these “stress hormones” [19,20]. Drug seeking could also then be viewed as a means for maintaining critical neurotransmitter pools for individuals who genetically have elevated tone or a “high set point” for each of these three catecholamines [19,20].

There may be several other consequences if the hypothesis put forth here is verified. It may suggest that methylation and demethylation are actually important and common processes in regulating organic molecules in addition to DNA, in the body. An additional point is that it is not obvious to the author that other major types or classes of drugs, such as marijuana and nicotine (or caffeine), are also acutely converted to catecholamines or other endogenous signaling molecules. Inspection of the molecular structures of THC (i.e., the active ingredient in marijuana) and nicotine, for example, does not reveal the same moieties as in the various stimulants, opioids, and psychedelics described above. THC and nicotine may, however, be acutely transformed to catecholamines through other molecular mechanisms. A final point is that many of the drugs described above occur in nature, whereas others appear not to (and are synthetically manufactured). It is nonetheless suggested here that the body has evolved molecular mechanisms, including various currently unidentified enzymes, for transforming all of these drugs to dopamine, norepinephrine, and epinephrine. One psychedelic drug discussed above, DMT, is actually present endogenously in the mammalian brain, including in rodents [21].

As noted above, this publication is suggesting that biotransformation of various drugs of abuse to catecholamines is occurring in addition to the known reactions of these drugs to form their conventional, already identified metabolites. In this scenario, only a perhaps small subset of these drug molecules is acutely transformed to catecholamines, whereas most of these drug molecules are rapidly converted to known metabolites through previously demonstrated pharmacokinetic pathways. A large number of prior publications have identified the metabolites of, for example, cocaine, amphetamine, heroin, morphine, LSD, DMT, and ethanol, where a number of these metabolites themselves have psychotropic qualities [22,23,24,25,26,27]. This publication is not suggesting that those various metabolic pathways are incorrect, but rather that a perhaps minor subset of those drug molecules is instead converted to catecholamines in a large enough quantity to nonetheless boost sympathetic and HPA axis signaling.

While the overall hypothesis put forth in this publication is very general in covering a broad range of drugs, sympathetic nervous system and HPA axis activation are also known to broadly accompany the effects of these various classes of drugs, providing a rationale for tying together transformation to catecholamines with the different classes in this hypothesis-based publication. Additionally, the various classes of drugs contain similar molecular moieties, suggesting some degree of plausibility that they could be transformed in similar ways in vivo. One possibility, however, is that only a subset of these drugs is converted to catecholamines in the body. It is moreover not clear that all of the proposed biochemical reactions, including in vivo demethylation of hydrocarbons, are thermodynamically plausible. It is also, as noted above, not known which enzymes may catalyze these proposed novel reactions, and whether such putative enzymes could have evolved in various organisms. These questions remain to be addressed by future molecular experimentation, in conjunction with the stable isotope methodology described here.

In conclusion, this paper has put forth the hypothesis that a wide range of drugs of abuse—stimulants, opioids, psychedelics—are acutely transformed to catecholamines in vivo. This may partially explain the stimulatory effect of these drugs on sympathetic nervous system and HPA axis signaling, as well as modulation of their subjective psychotropic properties, while suggesting a potential underlying role in substance abuse and dependence. Importantly, relatively simple experiments can be conducted, involving administration of stable isotope versions of these drugs to model organisms followed by liquid chromatography-mass spectrometry, to test whether portions of the drugs are indeed incorporated into new dopamine, norepinephrine, and epinephrine molecules.

## Data Availability

Not applicable.
